# Significant decrease of saturation index in erythrocytes membrane from subjects with non-alcoholic fatty liver disease (NAFLD)

**DOI:** 10.1186/s12944-017-0552-0

**Published:** 2017-08-23

**Authors:** Maria Notarnicola, Maria Gabriella Caruso, Valeria Tutino, Caterina Bonfiglio, Raffaele Cozzolongo, Vito Giannuzzi, Valentina De Nunzio, Giampiero De Leonardis, Daniela I. Abbrescia, Isabella Franco, Vincenza Intini, Antonella Mirizzi, Alberto R. Osella

**Affiliations:** 1 National Institute of Gastroenterology “S. de Bellis” Research Hospital, 70013 Castellana Grotte, Bari, Italy; 2 Laboratory of Nutritional Biochemistry, National Institute of Gastroenterology “S. de Bellis”, Research Hospital, Castellana Grotte, Bari, Italy

**Keywords:** Lipidomic analysis, Fatty acids profile, NAFLD, Erythrocyte membrane, Saturation index

## Abstract

**Background:**

The lipidomic profiling of erythrocyte membranes is expected to provide a peculiar scenario at molecular level of metabolic and nutritional pathways which may influence the lipid balance and the adaptation and homeostasis of the organism. Considering that lipid accumulation in the cell is important in promoting tissue inflammation, the purpose of this study is to analyze the fatty acid profile in red blood cell membranes of patients with Non-Alcoholic Fatty Liver Disease (NAFLD), in order to identify and validate membrane profiles possibly associated with the degree of hepatic damage.

**Methods:**

This work presents data obtained at baseline from 101 subjects that participated to a nutritional trial (registration number: NCT02347696) enrolling consecutive subjects with NAFLD. Diagnosis of liver steatosis was performed by using vibration-controlled elastography implemented on FibroScan. Fatty acids, extracted from phospholipids of erythrocyte membranes, were quantified by gas chromatography method.

**Results:**

The subjects with severe NAFLD showed a significant decrease of the ratio of stearic acid to oleic acid (saturation index, SI) compared to controls, 1.281 ± 0.31 vs 1.5 ± 0.29, respectively. Low levels of SI in red blood cell membranes, inversely associated with degree of liver damage, suggest that an impairment of circulating cell membrane structure can reflect modifications that take place in the liver. Subjects with severe NAFLDalso showed higher levels of elongase 5 enzymatic activity, evaluated as vaccenic acid to palmitoleic acid ratio.

**Conclusions:**

Starting from these evidences, our findings show the importance of lipidomic approach in the diagnosis and the staging of NAFLD.

## Background

The maintenance of physiological cell membrane fluidity is a prerequisite associated with normal cell growth and division [1] and its alterations have been described in various pathologies, such as obesity, diabetes mellitus and different types of cancer [1–4]. Lipid accumulation in the cell is important in promoting tissue inflammation [5] and this damage is not caused not only by the quantity but also by the quality of accumulated lipids in the cells [6]. In this contest, lipidomic analysis, as a new technology, allows to identify specific metabolic profiles, typical of a disease, as NAFLD.

The lipidomic profiling of erythrocyte membranes provides exhaustive information on metabolic and nutritional pathways which influence the lipid balance and homeostasis of the organism. Several studies have demonstrated that the analysis of fatty acids composition of circulating erythrocyte membrane can be considered an appropriate biomarker for investigating the relations between the patterns of fatty acid metabolism and specific diseases [7, 8], considering that changes in fatty acids composition of erythrocyte membranes may be related to illness status.

Previously, we showed the presence of an altered fatty acid profile in erythrocyte membranes of patients with colorectal cancer [9]. The patients suffered of colorectal cancer showed a lower percentage of total *n*-3-series polyunsaturated fatty acids (n-3-PUFAs) than controls and consequently this finding was reflected in the higher ratio n-6-PUFA/n-3-PUFA observed in patients having cancer compared to controls.

The saturation index (SI) of cell membrane is the ratio of stearic acid to oleic acid and it is considered an indicator of membrane rigidity [7]. Low levels of SI have been associated to the cell malignant phenotype [3]. Analysis of fatty acid extracted from leukemic cells and solid tumor tissue showed a significant increase in the oleic acid content respect to stearic acid [10, 11].In erythrocytes membranes, low levels of SI have been demonstrated to be predictors of postmenopausal breast cancer [12].

A lipidomic approach has been considered for the detection of the major lipid classes and for the distribution of fatty acids in cell membrane in experimental models of NAFLD and Non-alcoholic Steato-Hepatitis (NASH) [5, 13]. A modified lipidomic profile has been observed in NAFLD, suggesting that changes in the concentration and quality of lipids in cell membranes might affect the pathogenesis and progression of steatosis or steatohepatitis. Furthermore, NAFLD has been associated with an increase in saturated fatty acids altering the hepatic lipid profile in patients with liver damage [5, 6].

Therefore, considering that the changes in the concentration and quality of fatty acids play a central role in the development and progression of NAFLD, the present study was designed to analyze the fatty acids profile in red blood cell membranes of patients with NAFLD in order to identify and validate membrane profiles associated with hepatic injury. Moreover, a possible clinical association between specific lipidomic profiles and degree of liver damage will be assessed.

## Methods

### Patients

This work is part of NUTRIATT (NUTRItion and AcTiviTy) study, a nutritional trial (registration number: NCT02347696) enrolling consecutive subjects with NAFLD. Diagnosis of NAFLD was performed by using vibration-controlled elastography (VCTE) implemented on FibroScan® (Echosens, Paris, France.) [14]. NAFLD was categorized as absent (<215 dB), mild (215–250 dB), moderate (251–299 dB) and severe (≥300 dB) [15].

Trial inclusion criteria included: Body Mass Index (BMI) ≥ 25, calculated as weight in kilograms divided by the square of the height in meters (kg/m^2^); age > 30 years old and <60 years old; NAFLD moderate or severe. Exclusion criteria included: overt cardiovascular disease and revascularization procedures; stroke; clinical peripheral artery disease; current treatment with insulin or oral hypoglycemic drugs; fasting glucose >126 mg/ dl, or casual glucose >200 mg/dl; more than 20 g/daily of alcohol intake; severe medical condition that may impair the person to participate in a nutritional intervention study; people following a special diet or involved in a program for weight loss, or who had experienced recent weight loss and inability to follow a diet for religious or other reasons. The assessment of alcohol intake was performed by means of a questionnaire [16] and a personal interview.

In this study, we present data obtained at baseline from 101 subjects (55 males and 46 females) enrolled consecutively in NUTRIATT study. Participants were fasted for 12 h prior to examination. Blood samples taken from the subjects by venous puncture were collected in tubes containing Ethylenediamine tetraacetic Acid (K-EDTA) anticoagulant.

For in vitro isolation of erythrocytes, blood samples with K-EDTA were quickly layered on a Ficoll-Paque solution and centrifuged at 400×*g* for 40 min at 20 °C. The lymphocytes and plasma were then removed and the erythrocytes were recovered from the bottom layer and washed with 4-volumes of phosphate-buffered saline. Isolated red blood cells were stored at −80 °C until they were assayed. Moreover, aliquots of blood serum were taken from each subject and shipped to the central laboratory for routine analyses. All the analyses were performed within 3 months.

All subjects gave their informed consent to participate in the study.

### Fatty acids extraction, purification and preparation of fatty acid methyl esters

We used the modified method of Moilanen [17], that is itself a modification of the method described by Folch [18]. Each sample of red blood cells (RBC) was thawed bringing to room temperature. Fatty acids were hydrolyzed from phospholipids of RBC membranes by adding 0.9 ml of an acidified salt solution (H_2_SO_4_ 2·10^−4^ M, NaCl 0.1%). Afterwards, were added 5.0 ml of chloroform: methanol (2:1, *v*/v) (Sigma-Aldrich, Milan, Italy) and the samples were mixed thoroughly and centrifuged at 1000 × *g* for 10 min. The lower layer, containing fatty acids, was removed with care, replaced in a new tube and dried by a centrifugal evaporator (Bio-Rad, Milan, Italy). Preparation of fatty acid methyl esters (FAME) was carried out by adding 1 ml of toluene and 1.5 ml of BF_3·_MeOH 14% (Sigma-Aldrich, Milan, Italy) and incubating for 2 h at 80 °C. To the samples were added 2.5 ml of 5% aqueous sodium chloride solution, 1.5 ml of toluene and then centrifuged at 470×*g* for 10 min. The upper layer, containing FAME, was collected and transferred into a vial and analyzed.

### Gas chromatography and fatty acids quantification

Fatty acids quantification was performed by using a gas chromatography equipment with auto-sampler, a split/split less injector, FID detector and a hydrogen gas generator (Thermo Fisher Scientific, Milan, Italy). Separation of FAME was carried out on a BPX 70 capillary column SGE Analytical Science, P/N SGE054623, 60 m × 0.25 mm ID – BPX70 0.25 UM (SGE Europe Ltd., United Kingdom). Hydrogen was used as carrier gas, 3.0 ml min^−1^, constant flow mode; the amount injected was 1 μl in split less mode (split flow 50 ml min^−1^, split less time 1 min). The temperature of the injector and the FID detector were 250 °C. The initial temperature of the oven was 40 °C, then it increased to 170 °C at 10 °C min^−1^ for 5 min, then to 200 °C at 4 °C min^−1^ for another 5 min and finally the temperature increased to 240 °C at 10 °C min^−1^and held for 5 min.

Quantification of fatty acid methyl esters was performed using a mixture of standards (Supelco 37-Component FAME Mix, Sigma-Aldrich, Milan, Italy).

### Statistical analysis

All data were expressed as mean ± SD. ANOVA and Tukey Multiple comparison test were performed to estimate differences among groups. A probabilistic type I error of ≤0.05 was considered as statistically significant.

Multivariate regression analysis was performed to evaluate the role of confounding factors on final results. All analysis were performed by using Stata 14.2 statistical software.

## Results

Table 1 shows clinical and biochemical characteristics of subjects at the baseline stratified for degree of NAFLD. We found 24 subjects with absentNAFLD (i.e. control group), 30 with moderate and 47 with severe NAFLD.

**Table 1:** Clinical and biochemical variables in controls and patients with NAFLD

	Controls (*n* = 24) mean ± SD	Moderate NAFLD (*n* = 30) mean ± SD	Severe NAFLD (*n* = 47) mean ± SD	*P* value
Age	50.8 ± 8.4	50.5 ± 10.6	50.5 ± 10.6	0.4
BMI	30.9 ± 3.2	30.7 ± 2.9	32.3 ± 4.9	0.02 Controls vs Severe
Azotemia	21.5 ± 21.5	36.4 ± 10.2	34.6 ± 9.8	0.001 Controls vs Moderate and Severe
Ferritin	114.7 ± 148.6	137.9 ± 142.2	149.4 ± 178.9	0.42
ALT	28.9 ± 20.4	29.2 ± 10.3	40.7 ± 23.0	0.02 Controls vs Severe
AST	21.91 ± 4.18	23.53 ± 4.91	29.37 ± 10.84	0.01 Controls vs Severe
GGT	29.9 ± 46.5	23.5 ± 12.1	31.9 ± 28.3	0.8
Total cholesterol	198.6 ± 33.3	210.6 ± 33.9	208.6 ± 35.9	0.28
Cholesterol- HDL	51.6 ± 13.2	48.1 ± 13.7	41.1 ± 8.3	0.001 Controls vs Severe
Triglycerides	100.7 ± 46.5	118.7 ± 69.1	151.2 ± 89.0	0.01 Controls vs Severe
Glycemia	87.4 ± 12.2	93.2 ± 6.0	98.8 ± 15.1	0.001 Controls vs Severe
Serum Insulin	7.18 ± 3.03	9.65 ± 3.96	13.2 ± 4.8	0.04 and 0.001 Controls vs Moderate and Severe, respectively
HOMA index	1.59 ± 0.72	2.22 ± 0.90	3.16 ± 1.27	0.04 and 0.001 Controls vs Moderate and Severe, respectively
SFAs	52.6 ± 8.8	52.3 ± 9.6	53.1 ± 10.9	0.84
MUFAs	22.4 ± 5.5	22.6 ± 6.2	22.0 ± 7.2	0.8
PUFAs	24.9 ± 5.7	25.0 ± 4.6	24.8 ± 5.6	0.9

To probe the consistency and validity of our estimates we performed a post-hoc sample size estimation by taking into account our effect estimates. Type I (α) and II (β) probabilistic errors varied between 0.05 and 0.01 and between 0.20 and 0.05 respectively, so the power of the study varied from 0.80 to 0.95. Sample sizes obtained varied from *n* = 4 (α = 0.05, power 0.80) to *n* = 30 (α = 0.01, power 0.95) and then, the number of samples resulted appropriate.

The values of serum azotemia, ferritin, triglycerides, cholesterol-HDL, glycemia, insulin and HOMA index were significantly higher in the subjects with NAFLD compared to control group. Moreover, stratifying for score of NAFLD, a significant trend from lower to higher degree of NAFLD for these parameters was also detected (Table 1).

**Fig. 1:**
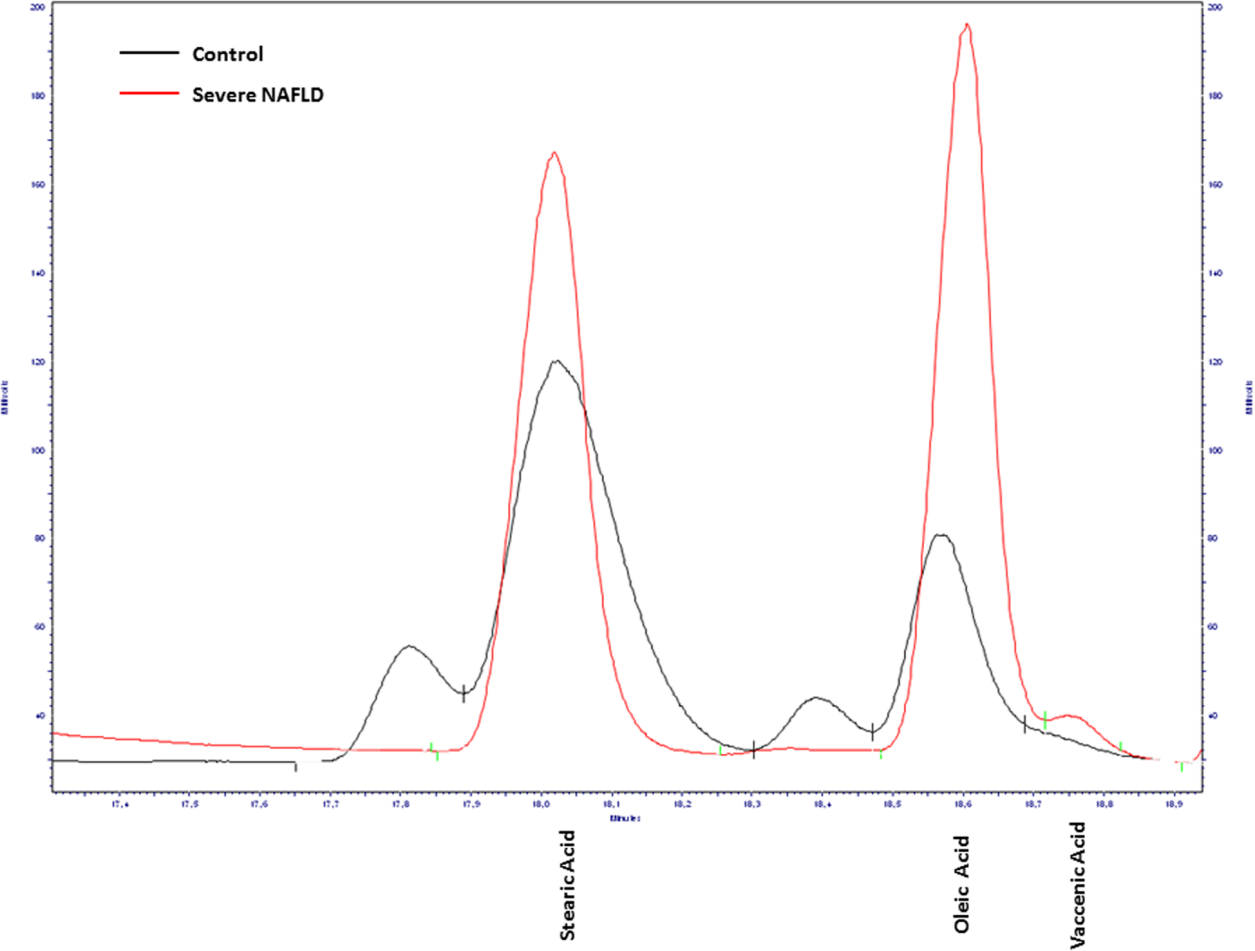
Chromatogram profile of fatty acids composition of erythrocytes membrane from a control subject and patient with severe NAFLD

Figure 1 shows a representative profile of fatty acids composition of erythrocytes membrane from a subject with severe NAFLD and a subject without liver injury.

**Fig. 2:**
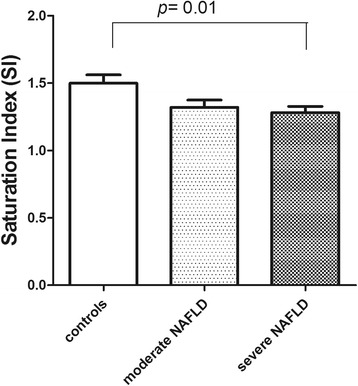
Saturation index (SI) levels (stearic acid/oleic acid ratio) in erythrocytes membranes from controls and patients with moderate and severe NAFLD. Data are expressed as the mean ± SD. *P <* 0.05, one-way analysis of variance and Tukey’s multiple comparison test

A significant decrease of the erythrocyte membrane SI was observed in subjects with severe NAFLD compared to controls, 1.281 ± 0.31 vs 1.5 ± 0.29, (Fig. 2, *p* = 0.01, ANOVA and Tukey Multiple comparison test). Lowering of SI was essentially due to the increase of oleic acid content detected in these samples.

**Fig. 3:**
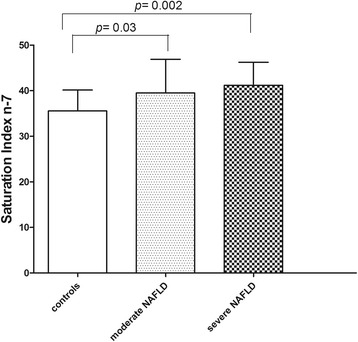
Saturation index n-7 levels (palmitic acid/palmitoleic acid ratio) in erythrocytes membranes from controls and patients with moderate and severe NAFLD. Data are expressed as the mean ± SD. *P <* 0.05, one-way analysis of variance and Tukey’s multiple comparison test

**Fig. 4:**
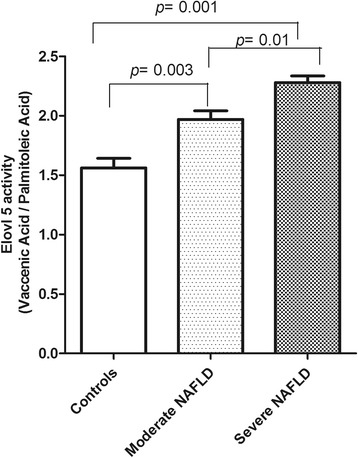
Levels of Elongase 5 (Elovl5) enzymatic activity in erythrocytes membranes from controls and patients with moderate and severe NAFLD. Data are expressed as the mean ± SD. *P <* 0.05, one-way analysis of variance and Tukey’s multiple comparison test

On the other hand, compared to controls, the subjects with severe NAFLD showed higher levels of SI n-7, defined as palmitic to palmitoleic acid ratio (Fig. 3, *p* < 0.05, ANOVA and Tukey Multiple comparison test). Moreover, the subjects with severe NAFLD reported enhanced levels of elongase activity, showing high levels of vaccenic acid (Fig. 1), a product derived from palmitoleic acid through the enzymatic activity of elongase 5 (Elovl5), (Fig. 4, *p*<0.05, ANOVA and Tukey Multiple comparison test).

No significant difference, among groups, was observed in fatty acids profile regarding total Saturated Fatty Acids (SFAs), Monounsaturated Fatty Acids (MUFAs) and PUFAs levels.

To evaluate the role of confounding factors on final results, we performed a multivariate regression analysis (Table 2). In the subjects with moderate and severe NAFLD, SI value was confirmed to be significantly lower respect to controls. This findings supports a role of SI as a biomarker of liver damage.

**Table 2:** Multivariate Regression Analysis: Effect of NAFLD score on red blood cell lipid profile

	SFAs	MUFAs	PUFAs	SI	SI n-7	Elovl5
	(95% C.I.)	(95% C.I.)	(95% C.I.)	(95% C.I.)	(95% C.I.)	(95% C.I.)
Moderate NAFLD	−2.07	0.25	1.82	−0.65*	16.81	0.64
	(−8.51,4.37)	(−3.92,4.41)	(−1.66,5.30)	(−1.19,-0.11)	(−19.49,53.11)	(−5.66,6.94)
Severe NAFLD	−1.54	−0.59	2.089	−0.54*	25.56	−0.20
	(−7.86,4.85)	(−4.71,3.52)	(−1.34,5.52)	(−1.08,-0.01)	(−10.26,61.38)	(−6.41,6.02)
Age (years)	0.23	−0.17	−0.065	0.01	−0.76	0.08
	(−0.06,0.52)	(−0.36,0.02)	(−0.22,0.09)	(−0.02,0.03)	(−2.41,0.90)	(−0.02,0.37)
Gender (Female)	15.34	−8.20	−7.110	−0.25	−0.40	3.83
	(−6.95,37.64)	(−22.64,6.22)	(−19.15,4.93)	(−2.13,1.62)	(−126.03,125.24)	(−17.98,25.63)

(95% C.I.) * *p* < 0.05; ^Adjusted for Age, Gender and biochemical markers

## Discussion

The clinical importance of NAFLD is linked to evidence that it might evolve in steatohepatitis, liver cirrhosis and cancer [19]. In the effort to detect novel and non-invasive biomarkers for diagnosis of NAFLD, this study demonstrates a relationship between alterations of circulating cell membrane lipidomic profile and degree of liver injury. Lower levels of SI in red blood cell membranes, detected in the patients with severe NAFLD, suggest an impairment of circulating cell membrane structure that can reflect modifications that take place in the liver.

It is widely known that in the pathophysiology of NAFLD, an elevated content of lipids in hepatic tissue, is a major cause of liver damage, affecting cell membrane structure [20].

In this context, we found the SI in red blood cells to be inversely associated with degree of liver damage. This finding, was also confirmed by multivariate regression analysis, supporting a role of SI as a biomarker of liver injury.

Elevated levels of oleic acid in erythrocytes membrane and consequently low levels of SI have been demonstrated in different pathological conditions [8, 21]. Low levels of SI have been associated with an increased cancer risk [3, 8], suggesting that the regulation of membrane fluidity is essential for the metabolic functionality of cells. The reduction of membrane rigidity seems to lead to increased cell metabolism and to higher cell proliferation activity, both features of neoplastic cells.

In this study, we showed that the ratio of saturated to unsaturated fatty acids can be a marker of liver damage and indirectly that the maintenance of normal oleic acid content might preserve the fluidity of membranes, a key feature of cell homeostasis.

Several studies have demonstrated that in the pathogenesis of NAFLD, an altered regulation of desaturation and elongation processes of fatty acids are present. High levels palmitic to palmitoleic acid ratio and lower levels of oleic acid to stearic acid ratio, observed in patients with NAFLD, are probably due to an increased chain elongation and to an altered control of desaturase signaling. A key role in these enzymatic processes is played by stearoyl-coenzyme A desa turase 1, an important enzyme of triglycerides biosynthesis, specific lipid class accumulated in livers of subjects with NAFLD [22].

Low content of palmitoleic acid, detected in red blood cells membranes of the subjects with severe NAFLD, was justified by high levels of elongase 5 (Elovl5) activity. Elevated activity of Elovl5 enzyme leads to a higher transformation rate of palmitoleic acid in vaccenic acid. Regarding C18:1 n-7, likely *cis*- vaccenic acid, a recent study suggested a role for this fatty acid in the development of chronic kidney disease [23]. Moreover, circulating concentrations of *cis*-vaccenic acid have been demonstrated to be associated with an increased risk of coronary heart disease and cardiac arrest [24, 25].

Consistently with our data, being NAFLD associated with cardiovascular diseases [26], high levels of *cis*-vaccenic acid, correlated with higher degree of liver damage detected in this study, confirm the role for specific fatty acids species in the onset of metabolic pathologies.

## Conclusions

We conclude that the lipidomic approach is important in the diagnosis and the staging of NAFLD. Our data, overall, suggest that SI could be used as a biomarker of liver injury in order to assess therapeutic intervention to restore membrane fatty acids profile. Changes in the dynamics of the fatty acid metabolic processes through diet and life style can be beneficial for the patients with this metabolic disease.

## References

[1] Habib NA, Wood CB, Apostolov K, Barker W, Hershman MJ, Aslam M, Heinemann D, Fermor B, Williamson RCN, Jenkins WE, Masters JRW, Embleton MJ (1987). Stearic acid and carcinogenesis. Br J Cancer.

[2] Cooper RA (1977). Abnormalities of cell-membrane fluidity in the pathogenesis of disease. N Engl J Med.

[3] Wood CB, Habib NA, Thompson A, Bradpiece H, Smadja C, Hershman M, Barker W, Apostolov K (1985). Increase of oleic acid in erythrocytes associated with malignancies. Br Med J.

[4] Persad RA, Gillatt DA, Heinemann D, Habib NA, Smith PJ (1990). Erythrocyte stearic to oleic acid ratio in prostatic carcinoma. Br JUrol.

[5] Puri P, Baillie RA, Wiest MM, Mirshahi F, Choudhry J, Cheung O, Sargeant C, Contos JC, Sanyal AJ (2007). A lipidomic analysis of non-alcoholic fatty liver disease. Hepathology.

[6] Serviddio G, Bellanti F, Villani R, Tamborra R, Zerbinati C, Blonda M, Ciacciarelli M, Poli G, Vendemiale G, Iuliano L (2016). Effects of dietary fatty acids and cholesterol excess on liver injury: a lipidomic approach. RedBiol.

[7] Pala V, Krogh V, Muti P, Chajès V, Riboli E, Micheli A, Saadatian M, Sieri S, Berrino F (2001). Erythrocyte membrane fatty acids and subsequent breast cancer: a prospective Italian study. J Natl Cancer Inst.

[8] Pandey M, Sharma LB, Singh S, Shukla VK (2003). Erythrocyte membrane fatty acid profile and saturation index in gallbladder carcinogenesis: a case-control study. World JSurgOncol.

[9] Coviello G, Tutino V, Notarnicola M, Caruso MG (2014). Erythrocyte membrane fatty acids profile in colorectal cancer patients: a preliminary study. Anticancer Res.

[10] Apostolov K, Barker W, Catovsky D, Goldman J, Matutes E (1985). Reduction in stearic to oleic in leukaemic cells: a possible chemical marker of malignancy. Blutalkohol.

[11] Wood CB, Habib NA, Apostolov K, Thompson A, Smadja C, Hershman M, Baker W (1985). Reduction in the stearic to oleic acid ratio in circulating red blood cells: a possible tumor marker in solid human neoplasm. Eur J Surg Oncol.

[12] Chajes V, Hulten K, Van Kappel AL, Winkvist A, Kaaks R, Hallmans G, Lenner P, Riboli E (1999). Fatty acid composition in serum phospholipids and risk of breast cancer: an incident case-control study in Sweden. Int J Cancer.

[13] Almeda-Valdes P, Altamirano-Barrera A, Mendez-Sanchez N (2015). Insights in non-alcoholic fatty liver disease pathophysiology with lipidomic analyses. Annals of Hepatol.

[14] Berzigotti A (2014). Non-invasive assessment of non-alcoholic fatty liver disease: ultrasound and transient elastography. Rev Rec Clin Trials.

[15] Berzigotti A (2014). Getting closer to a point-of-care diagnostic assessment in patients with chronic liver disease: controlled attenuation parameter for steatosis. JHepatol..

[16] Skinner HA, Sheu WJ (1982). Reliability of alcohol use indices. The lifetime drinking history and the MAST. J Stud Alcohol.

[17] Moilanen T, Nikkari T (1981). The effect of storage on the fatty acid composition of human serum. Clin Chim Acta.

[18] Folch J, Lees M, Sloane Stanley GH (1957). A simple method for the isolation and purification of total lipids from animal tissues. J BiolChem.

[19] Caldwell SH, Crespo CM (2004). The spectrum expanded: cryptogenic cirrhosis and the natural history of non-alcoholic fatty liver disease. JHepatol.

[20] Matsuzawa N, Takamura T, Kurita S, Misu H, Ota T, Ando H, Yokoyama M, Honda M, Zen Y, Nakanuma Y, Miyamoto K, Kaneko S (2007). Lipid-induced oxidative stress causes steatohepatitis in mice fed with an atherogenic diet. Hepatology.

[21] Kelly SB, Miller J, Wood CB, Williamson RCN, Habib NA (1990). Erythrocyte stearic acid desaturation in patients with colorectal carcinoma. Dis Colon Rectum.

[22] Yee JK, Mao CS, Hummel HS, Lim S, Sugano S, Rehan VK, Xiao G, Lee WNP (2008). Compartmentalization of stearoyl-coenzyme a desaturase 1 activity in HepG2 cells. J Lipid Res.

[23] Block R, Kakinami L, Liebman S, Shearer GC, Kramer H, Tasi M (2012). Cis-vaccenic acid and the Framingham risk score predict chronic kidney disease: the multi-ethnic study of atherosclerosis (MESA). Prostagl Leuk Essen Fatty Acid.

[24] Djoussè L, Matthan NR, Lichtenstein AH, Gaziano JM (2012). Red blood cell membrane concentration of cis-palmitoleic and cis-vaccenic acids and risk of coronary heart disease. Am J Cardiol.

[25] Wu JHY, Lemaite RN, Imamura F, King IB, Song X, Spiegelman D, Siscovick DS, Mozaffarian D (2011). Fatty acids in de novo lipogenesis pathway and risk of coronary heart disease: the cardiovascular health study. Am J Clin Nutr.

[26] Targher G, Byrne CD, Lonardo A, Zoppini G, Barbui C (2016). Nonalcoholic fatty liver disease and risk of incident cardiovascular disease: a meta-analysis of observational studies. J Hepatol.

